# 799 Autologous Skin Cell Suspension Versus Standard Split Thickness Autografting: a Comparison of Operative Efficiency

**DOI:** 10.1093/jbcr/irac012.348

**Published:** 2022-03-23

**Authors:** Kathryn Skibba, Derek Bell

**Affiliations:** University of Rochester, Rochester, New York; University of Rochester, Rochester, New York

## Abstract

**Introduction:**

The surgical standard of care (SOC) for deep burns is autologous grafting, which is currently challenged by an innovative technology, autologous skin cell suspension (ASCS). Sprayed epidermal autografting provides comparable results to SOC while reducing donor graft size and morbidity, making it ideal for patients with large total body surface area (TBSA).^1-3^ The purpose of this study is to report and compare the size of burn wound coverage per operative minute for ASCS versus standard split thickness autografting (STSG). The authors hypothesize that ASCS covers large burn wounds more efficiently than STSG.

**Methods:**

This study is a case-control analysis of all adult (age ≥18) surgical encounters for ASCS versus matched SOC STSG encounters at a single burn center. Patient demographics, burn characteristics, and surgical details were collected from the electronic medical record. Patients were matched with prioritization of treated size and anatomic area, followed by burn etiology, age, and sex.

**Results:**

Eleven ASCS surgeries were performed on 7 patients and matched to 11 STSG surgeries in 8 patients. The ASCS and STSG groups were not significantly different regarding age (46.6 vs 41.5), BMI (28.3 vs 26.4), and co-morbidities (57.1% vs 50.0%), respectively. Most burns resulted from flame injury (85.5% ASCS vs 87.5% STSG). Time to re-keratinization of the burn wound (20.4 vs 19.0 post-operative days; ASCS, STSG) and subjective analysis of graft adherence was comparable between matched groups.

Treated burn wounds were located on the lower extremity (63.6% vs 62.5%), upper extremity (27.3% vs 50%), and trunk (18.2% vs 25.0%) in ASCS and STSG encounters, respectively. The average graft thickness harvested was thicker for ASCS surgeries than STSG (0.0061″ vs 0.0054″, p= 0.040). The average burn wound size and operative duration was 2612.2 cm^2^ in 108.4 minutes for ASCS and 1903.7 cm^2^ in 92.1 minutes for STSG. As a result, ASCS covered 24.1 cm^2^ in comparison to STSG coverage of 20.7 cm^2^ per operative minute.

**Conclusions:**

The matched groups were not significantly different, and surgical encounters are acceptable for comparison. However, ASCS grafts were harvested thicker than STSG (0.0061″ vs 0.0054″, p= 0.040), which is unlikely to have any clinical implications as both are considered ultra-thin (< 0.008″).^5^

ASCS treats a larger wound size per operative minute than matched STSG controls. Our results are likely restricted to large burn wounds and may not translate to smaller treatment areas. The increased operative productivity with ASCS assists in reduction of the technology unit cost. In conclusion, selective use of ASCS for large burn wounds can partially offset the unit cost through increased operative efficiency, lessen donor site morbidity, and produce similar results as standard split thickness autografting.

